# Improved efficacy of an arthropod toxin expressing fungus against insecticide-resistant malaria-vector mosquitoes

**DOI:** 10.1038/s41598-017-03399-0

**Published:** 2017-06-13

**Authors:** Etienne Bilgo, Brian Lovett, Weiguo Fang, Niraj Bende, Glenn F. King, Abdoulaye Diabate, Raymond J. St. Leger

**Affiliations:** 1 Institut de Recherche en Sciences de la Santé/Centre Muraz, Bobo-Dioulasso, Burkina Faso; 2 Department of Entomology, University of Maryland, College Park, Maryland 20742 USA; 3 Institute of Microbiology, College of Life Sciences, Zhejiang University, Hangzhou, Zhejiang, 310058 China; 4 Institute for Molecular Bioscience, The University of Queensland, St. Lucia, QLD 4072 Australia

## Abstract

The continued success of malaria control efforts requires the development, study and implementation of new technologies that circumvent insecticide resistance. We previously demonstrated that fungal pathogens can provide an effective delivery system for mosquitocidal or malariacidal biomolecules. Here we compared genes from arthropod predators encoding insect specific sodium, potassium and calcium channel blockers for their ability to improve the efficacy of *Metarhizium* against wild-caught, insecticide-resistant anophelines. Toxins expressed under control of a hemolymph-specific promoter increased fungal lethality to mosquitoes at spore dosages as low as one conidium per mosquito. One of the most potent, the EPA approved Hybrid (Ca^++^/K^+^ channel blocker), was studied for pre-lethal effects. These included reduced blood feeding behavior, with almost 100% of insects infected with ~6 spores unable to transmit malaria within 5 days post-infection, surpassing the World Health Organization threshold for successful vector control agents. Furthermore, recombinant strains co-expressing Hybrid toxin and AaIT (Na^+^ channel blocker) produced synergistic effects, requiring 45% fewer spores to kill half of the mosquitoes in 5 days as single toxin strains. Our results identify a repertoire of toxins with different modes of action that improve the utility of entomopathogens as a technology that is compatible with existing insecticide-based control methods.

## Introduction

Some of the most important human diseases are borne by mosquitoes including malaria (*Anopheles*), filariasis (*Culex*, *Mansonia* and some *Anopheles* spp.), viral encephalitides (*Culex*), dengue and yellow fever (principally *Aedes aegypti*). An estimated 2 billion people live in areas where these diseases are endemic. The burden is heaviest in sub-Saharan Africa, where ~200 million cases of malaria are reported annually and many children succumb^1^. Emergence and spread of resistance to pyrethroids, organophosphates and carbamates is a particular threat, as most disease control programs rely heavily on these broad-spectrum chemical insecticides to reduce vector populations^2–5^. In recent years, anopheline malaria vectors in sub-Saharan Africa have gained sufficient resistance to render chemical insecticides largely ineffective^5, 6^.

The chemical insecticides used in mosquito control programs are directed at neuronal voltage-gated sodium (Na_V_) channels, as are many insect-specific neurotoxins derived from arthropods, including the scorpion toxin AaIT that has been approved for release in several countries^7^. A recombinant strain of the entomopathogenic fungus *Metarhizium anisopliae* expressing AaIT killed *Ae. aegypti* mosquitoes faster and at lower spore doses than wild-type (WT) fungus^8^. Since AaIT and pyrethroids bind to different sites on insect Na_V_ channels and channel mutations that confer resistance to pyrethroids actually increase binding of AaIT, the use of both pyrethroids and AaIT should mitigate against resistance acquisition^9^. Furthermore, combining toxins with different targets, such as voltage-gated sodium, potassium (K_V_) and calcium (Ca_V_) channels, could provide useful additive or synergistic effects and minimize the potential for cross-resistance. Ca_V_ and K_V_ channels are previously unexploited insecticide targets, reducing the likelihood of pre-existing resistance.

We compared the efficacy against *Anophel*es *gambiae* of recombinant *Metarhizium pingshaense* co-expressing green fluorescent protein (GFP) and one of several arthropod-derived toxins with different modes of action: AaIT, the Na_V_ channel inhibitor μ-CUTX-As1a (As1a)^10^, the K_Ca_/Ca_V_ blocker ω/κ-hexatoxin-Hv1a (Hybrid), the Ca_V_ channel blocker ω-hexatoxin-Hv1a (Hv1a)^11^, and U_1_-AGTX-Ta1a (Ta1a), a weaponized insect hormone which has an unknown neuronal target^12^. With the exception of AaIT, all these toxins are derived from spider venom. To express the transgenes we used the Mcl1 promoter that targets expression to the hemocoel of infected insects^8, 13, 14^.

We previously determined that hyphal bodies appear in the hemolymph 2–3 days post infection and that Mcl1 expression commences within 20 min of contact with hemolymph^15^.

**Figure 1:**
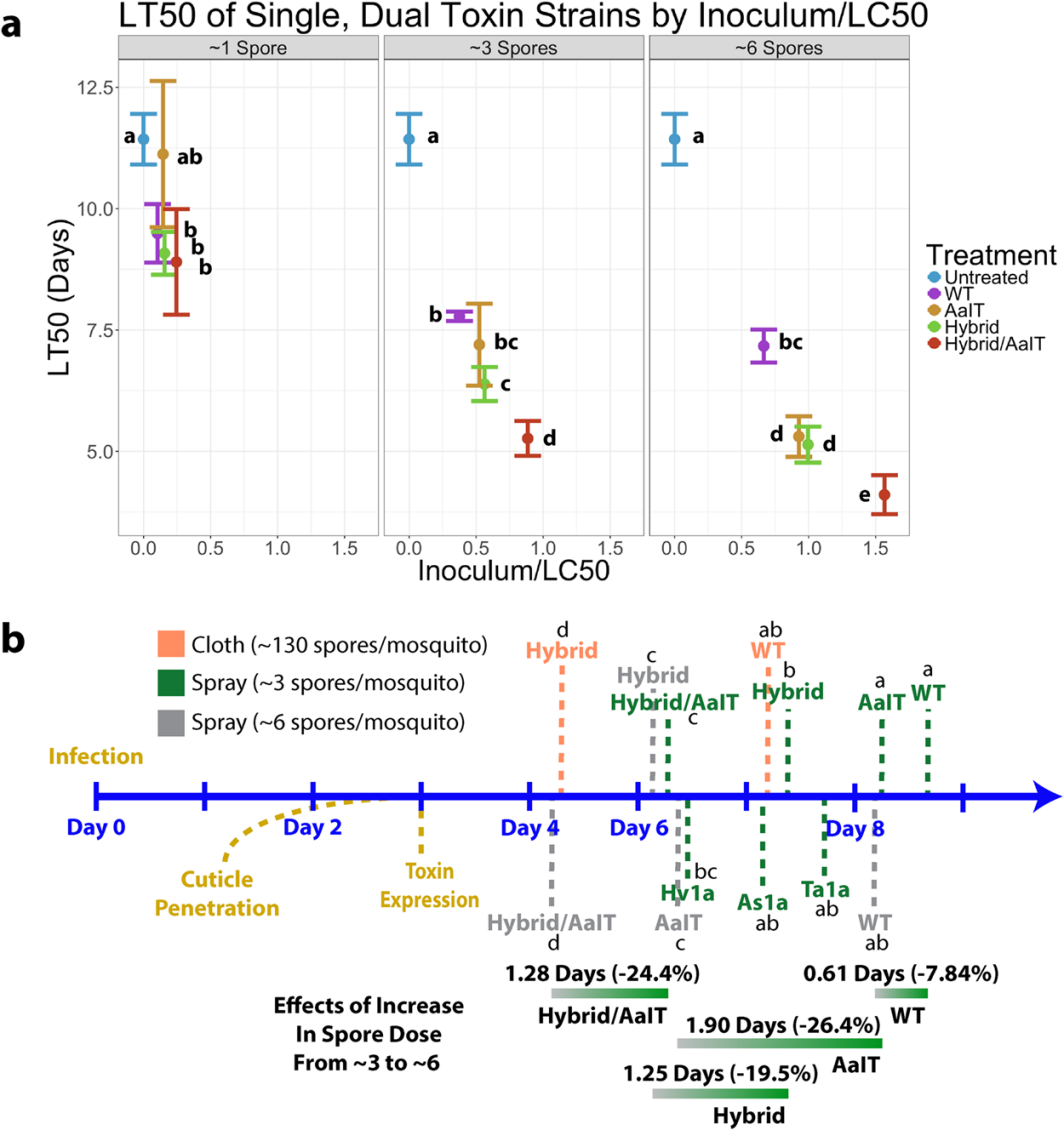
(**a**) LT50 values for mosquitoes treated with 1 × 10^5^, 1 × 10^6^ and 1 × 10^7^ *M. pingshaense* conidia/ml suspensions resulting in ~1, ~3 and ~6 conidia/mosquito, respectively, of *M. pingshaense* WT or M. *pingshaense* expressing Hybrid, AaIT or both AaIT and Hybrid. LC50 values are reported along the x-axis as the inverse of the estimated spore inoculum. Lettering represents statistical differences (p < 0.05) based on a log-rank test comparing the Kaplan-Meier survival curves. The LC50 dose for untreated mosquitoes was fixed at zero and reported for all spore concentrations for comparison. (**b**) Schematic representing infection timing (beige) and LT50s of mosquitoes treated with conidial suspensions and exposed to cloth impregnated with toxin expressing strains or WT. Lettering groups toxins by statistical significance (p < 0.05) based on a log-rank test comparing the Kaplan-Meier survival curves.

Transformants were selected by GFP expression and PCR/sequencing confirmation of the toxin gene, and initially bioassayed by spraying spore suspensions (~1 conidium/mosquito; ~3 conidia/mosquito; and ~6 conidia/mosquito) onto cold (4 °C) anesthetized adult female *An. gambiae*. Although they have different molecular targets (Na_V_, Ca_V_ and K_Ca_ channels), each toxin significantly (p < 0.05) improved the median lethal time (LT50) with infection loads of 3 or more spores (Fig. 1a,b, and Table 1) compared to WT, and therefore they constitute a potential arsenal that could be rotated and/or used in combination in a mosquito control program aimed at mitigating resistance. Hybrid, also known as Versitude^TM^, was chosen for further study as the US EPA has already approved it for use as a stand-alone insect control agent. Since prevention of transmission of malaria is of primary importance in assessing a mosquito control technology, we took a holistic view of the life cycle of the parasite to determine if pre-lethal effects could contribute to *Metarhizium* preventing the spread of malaria.

**Table 1:** L﻿T50s and LC50s (day 5) for *Anopheles gambiae* treated with *Metarhizium pingshaense* strains expressing arthropod-derived toxins alone and stacked (Hybrid/AaIT) compared with wild-type (WT) and a control (0.01% Tween).

Treatment	Statistic	Concentration conidia/mL	Mean (days)	SE (days)
**WT**	LT50	1.00E + 05	9.49	0.60
**WT**	LT50	1.00E + 06	7.78	0.10
**WT**	LT50	1.00E + 07	7.17	0.34
**AaIT**	LT50	1.00E + 05	11.1	1.51
**AaIT**	LT50	1.00E + 06	7.20	0.84
**AaIT**	LT50	1.00E + 07	5.30	0.42
**Hybrid**	LT50	1.00E + 05	9.08	0.44
**Hybrid**	LT50	1.00E + 06	6.39	0.35
**Hybrid**	LT50	1.00E + 07	5.14	0.37
**Hybrid/AaIT**	LT50	1.00E + 05	8.90	1.09
**Hybrid/AaIT**	LT50	1.00E + 06	5.27	0.36
**Hybrid/AaIT**	LT50	1.00E + 07	4.10	0.40
**As1a**	LT50	1.00E + 06	6.12	0.82
**Hv1a**	LT50	1.00E + 06	5.50	0.35
**Ta1a**	LT50	1.00E + 06	6.80	0.08
**Control**	LT50	NA	11.4	0.52
			conidia/mL	
**WT**	LC50	NA	1.69E + 07	3.72E + 06
**AaIT**	LC50	NA	1.09E + 07	1.90E + 06
**Hybrid**	LC50	NA	9.84E + 06	3.20E + 06
**Hybrid/AaIT**	LC50	NA	4.62E + 06	1.06E + 06

Mean and standard error is reported across three replicates.

We tested fungal efficacy against three anopheline vectors of human malaria: two wild-caught, insecticide-resistant species (*An. coluzzii* and *An. gambiae* s.s.) and one lab-reared, insecticide-susceptible species (*An. gambiae Kisumu An. kisumu*). We applied the fungus to sheets, as fungal-impregnated sheets hung in houses provide a resting area for mosquitoes that have taken blood meals^16^. Most malaria vectors prefer to blood feed and rest inside houses, thus maximizing the likelihood of fungal contact and infection^17^. We exposed mosquito populations for one hour to sheets impregnated with either: 1) Met-RFP, a strain with WT virulence expressing red fluorescent protein (RFP) as a marker, and 2) Hybrid toxin expressing fungus (Met-Hybrid) co-expressing GFP. We found mosquitoes exposed to the cloth for one hour in a WHO mosquito bioassay tube picked up an average of 129 ± 18 spores, a sufficient dose to kill all mosquitoes exposed to each fungal treatment. Mortality was monitored over 14 days and compared to uninfected controls.

Insecticide (Knockdown) resistance (*kdr*) in each mosquito population was quantified using PCR. The *kdr* mutation reduces sensitivity to DDT and pyrethroids and is the most prevalent form of insecticide resistance for West African mosquitoes^6^. The levels of *kdr* resistance in wild-caught *An. coluzzii* and *An. gambiae* s.s. were 98.3% and 92.9%, respectively (Supplementary Table 1). *An. gambiae* Kisumu mosquitoes are an established laboratory population of mosquitoes with stable susceptibility to insecticides, so their resistance was not tested.

**Figure 2:**
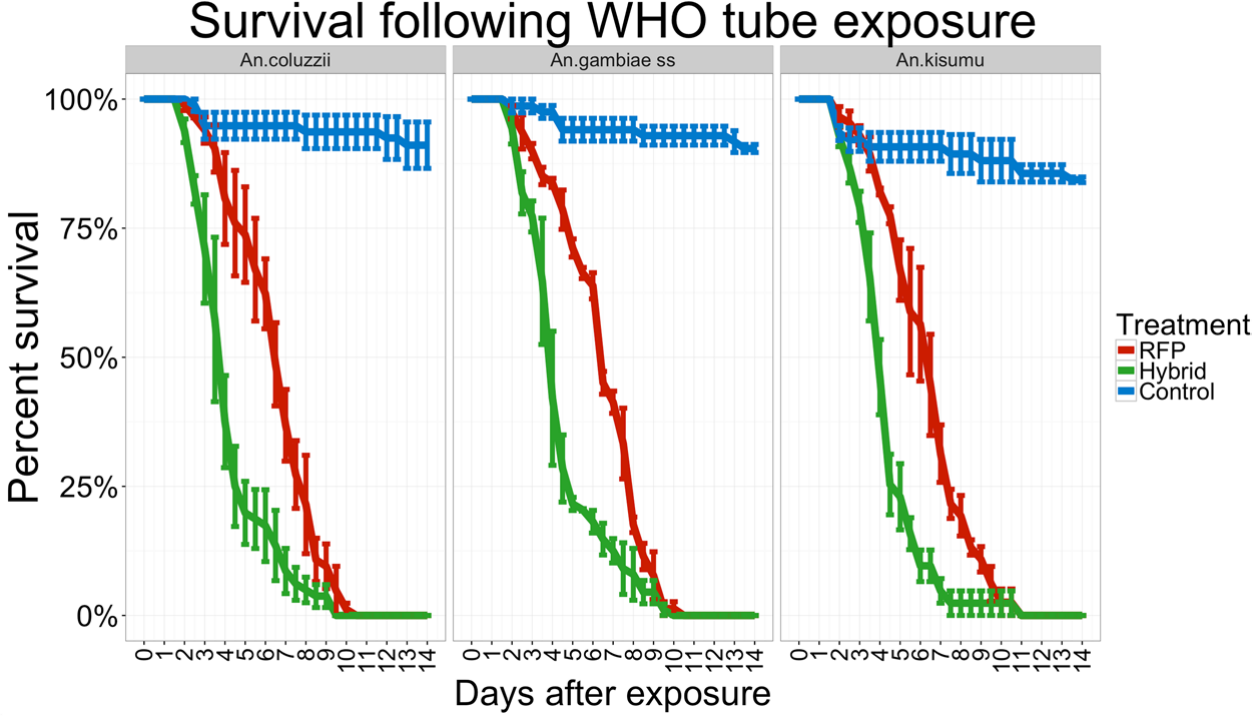
Survival following WHO tube exposure: these three graphs represent survival curves for RFP expressing *Metarhizium* (Met-RFP) and Hybrid toxin-expressing *Metarhizium* (Met-Hybrid) against two wild-caught, insecticide-resistant (*An. coluzzii* and *An. gambiae* s.s.) and one lab-reared (*An. gambiae Kisumu*) human malaria vector mosquitoes. There are no significant differences in time to kill insecticide resistant and susceptible strains, but Met-Hybrid is more effective on all mosquito strains.

Overall, insecticide resistance did not alter the susceptibility of the three mosquito species to Met-RFP and Met-Hybrid (Fig. 2). Within 2.5 days post-infection, mosquitoes exposed to Met-Hybrid were dying faster than those exposed to Met-RFP. LT50 (LT80) values for Met-Hybrid and Met-RFP were 4.14 ± 0.16 (5.47 ± 0.25) and 6.18 ± 0.14 (7.71 ± 0.16) days, respectively (mean ± standard error is reported). Fluorescent *Metarhizium* mycosis was observed on fungus-exposed cadavers, confirming mortality due to treatment. The number of mosquitoes surviving in the uninfected control group never dropped below 84.4% (Fig. 2).

The wild-type *M. pingshaense* strain has a narrow host range. To investigate if expressing Hybrid toxin impacts host specificity, we assayed transformants against honeybees (*Apis mellifera adansonii*) as a representative local pollinator. Honeybees were infected with black cloth impregnated with ~2 × 10^6^ conidia/cm^2^ in WHO tubes and spore dose was estimated using our selective media protocol. Met-RFP and Met-Hybrid did not kill any honeybees in the two weeks they were observed following infection (there was no difference in mortality rates between fungal exposed and unexposed bees, and no fungal emergence was observed on any bee cadaver). This is in agreement with previous studies indicating that the expression of insect toxins did not increase the host range of *M. acridum* ^13^. Interestingly, honeybees picked up significantly fewer spores (80 ± 3 spores, p < 0.05) than mosquitoes under the same conditions, despite the much smaller size of mosquitoes. Spraying bees with 1 × 10^8^ spores/ml also failed to cause mortality.

**Figure 3:**
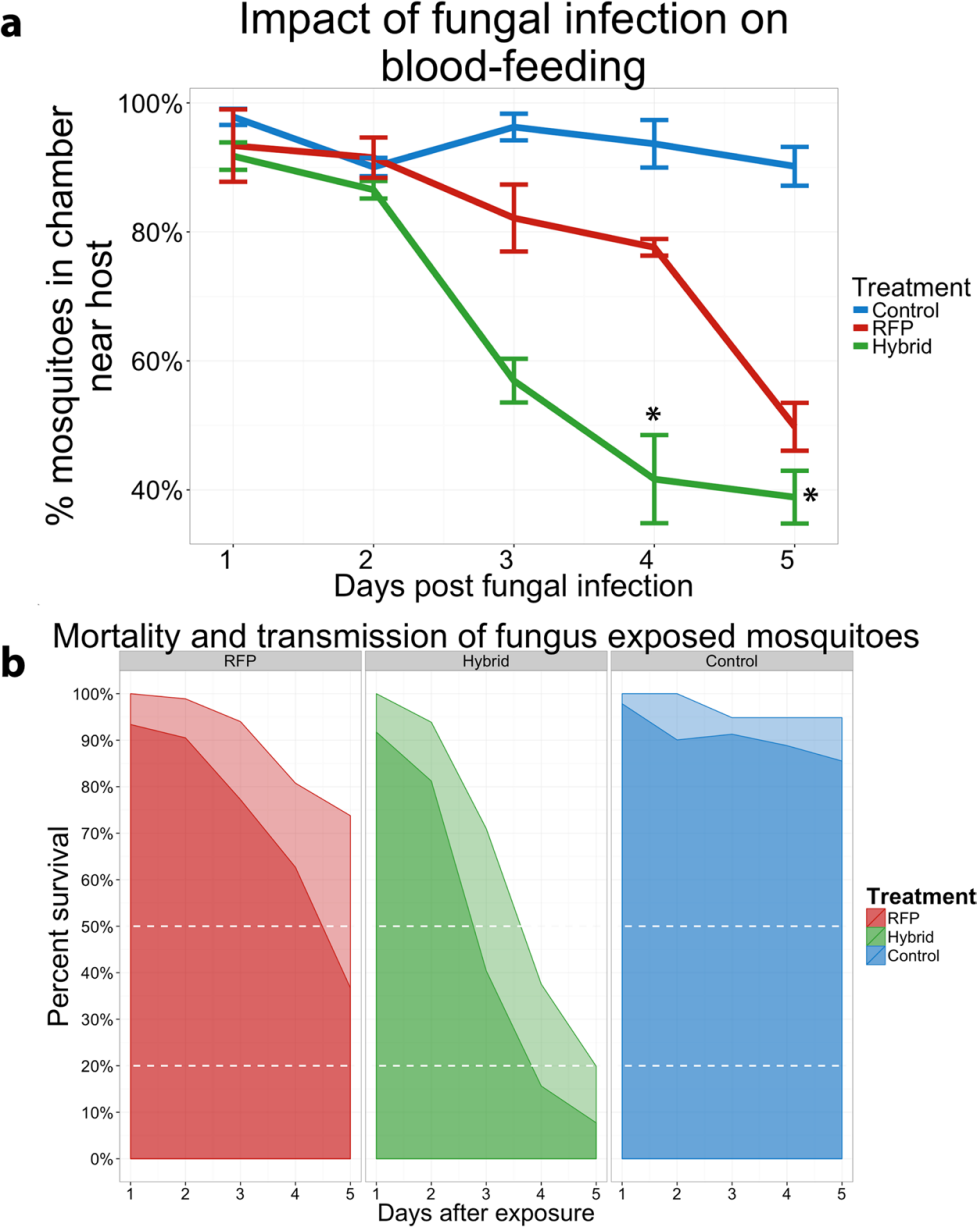
(**a**) Impact of fungal infection on blood-feeding at 1–5 days post-infection with either Met-Hybrid or Met-RFP. Mosquitoes were placed in a choice chamber with the guinea pig host outside of the chamber and just out of reach (Supplementary Figure 1a,b). Host-seeking (blood feeding) interest was quantified as the percentage of the mosquito population choosing the chamber closest to the host. The symbol “*” denotes no significant differences in mosquito choices with or without a host: 30 ± 3.05% of the mosquitoes chose the “host” chamber eve﻿n in the absence of a host. (**b**) Mortality and transmission of mosquitoes exposed to fungus, the light area represents the percent survival of mosquitoes for each treatment, while the dark area shows the proportion of mosquitoes in each treatment that are alive and would seek a host to blood feed (capable of malaria transmission). The upper dashed line represents the LT50 while the lower dashed line represents the 80% control threshold suggested by the World Health Organization (WHO) for a successful vector control agent^18^.

Willingness and ability to blood feed were also tested, as even small changes in coordination could potentially interfere with behavior and disease transmission. Host-seeking (blood feeding) interest was quantified as the percentage of the mosquito population choosing the chamber closest to the host in a guinea pig choice chamber (Fig. 3a,b and Supplementary Figure 1a,b). At one day post-infection, 94.3% of untreated (controls) and treated (Met-RFP or Met-Hybrid) mosquitoes flew toward the blood source with no significant differences between treatments. The willingness of mosquitoes in the control group to blood feed did not change over the course of the experiment. In contrast, significantly (p < 0.05) fewer (56.9%) mosquitoes treated with Met-Hybrid flew to the blood source on day 3 as compared to Met-RFP (82.1%). By day 4 the number of Met-Hybrid infected mosquitoes in the guinea pig choice chamber (38.9%) was not significantly different than the 30% entering the chamber in the absence of a guinea pig. Met-RFP infected mosquitoes only became significantly (p < 0.01) less responsive than uninfected controls on day 4 (Fig. 3a). These results suggest a pre-lethal advantage to using transgenic fungi for mosquito control.

With this information, we projected the measured proportion of mosquitoes interested in blood feeding onto the mortality of mosquitoes to identify the fraction capable of malaria transmission (Fig. 3b). In contrast to Met-RFP, by day 5, Met-Hybrid infected mosquitoes passed the threshold in both metrics (8.35% malaria transmission and >80% mortality) for the 80% control threshold suggested by the World Health Organization (WHO) for a successful vector control agent^18^.

Like Hybrid, AaIT is US EPA approved. As AaIT targets Na_V_ channels and the Hybrid toxin targets Ca_V_ and K_Ca_ channels, we examined whether their different sites of action produce synergistic effects. We found a clear benefit in terms of both effective spore doses and speed of kill in expressing both Hybrid and AaIT in a single strain (Met-Hybrid/AaIT) (Table 1). Mosquitoes sprayed with 1 × 10^5^ spores/mL (~1 spore/mosquito) of Met-Hybrid/AaIT, Met-Hybrid or WT had LT50s of 8.90 ± 1.09, 9.08 ± 0.44, and 9.49 ± 0.60 days, respectively, and the differences between them fell short of significance (Fig. 1a and Table 1). At this low spore dose, Met-Hybrid/AaIT, Met-Hybrid and WT significantly (p < 0.05) reduced lifespan compared to untreated mosquitoes (LT50 of 11.4 ± 0.52 days), and high variation in Met-AaIT bioassays resulted in no statistical difference from untreated mosquitoes. However, increasing the spore doses reduced the LT50s of toxin expressing strains to a greater extent than WT, consistent with the toxins having threshold effects (Fig. 1a,b). Comparing toxins, mosquitoes sprayed with 1 × 10^6^ Met-Hybrid/AaIT spores/ml (~3 spores/mosquito) achieved an LT50 of 5.30 ± 0.42 days that was significantly faster than the 6.39 ± 0.35 and 7.20 ± 0.84 days for Met-Hybrid and Met-AaIT alone. The Tabashnik synergism equation^19^ confirmed synergistic interactions as early as 4.5 days post-infection, and at 5 days it takes less than half the dosage of Met-Hybrid/AaIT to kill mosquitoes at the same rate as either Met-Hybrid or Met-AaIT applied alone (Supplementary Table 2). Such synergies suggest that optimizing the overall efficacy of the control strategy will require multiple transgenes, and a toxin arsenal could reduce effective conidial doses thereby reducing end-user costs (Fig. 1a and Table 1).

**Figure 4:**
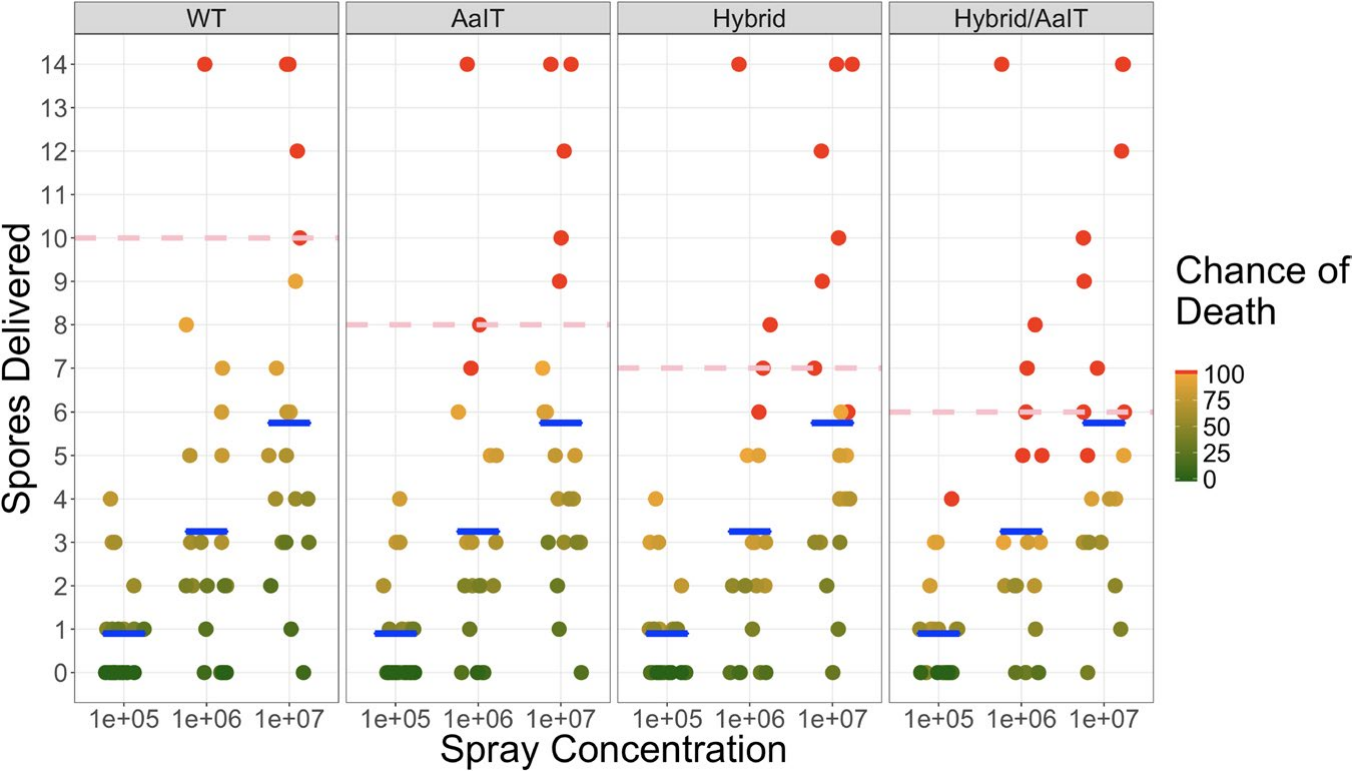
The number of spores infecting each mosquito after spraying with three different spore concentrations (1 × 10^5^, 1 × 10^6^, and 1 × 10^7^ spores/mL in 0.01% Tween80) plotted against the probability of death. The mean number of spores delivered with each suspension is marked with a blue crossbar. Assuming that mosquitoes with a higher dose are more likely to die, we calculated the chance of death for each mosquito based on our survival data for each treatment at each concentration. Mosquitoes with an estimated 100% chance of death are colored in red, and 0–99% is represented with a green to orange gradient. The red dashed line indicates the estimated LD100 in each treatment (10 spores for WT, 8 spores for AaIT, 7 spores for Hybrid and 6 spores for Hybrid/AaIT).

The spray infection method successfully probes efficacy differences between transgenes by delivering low spore doses, but contributes to variation in LT50 values because some mosquitoes escape infection at these low dosages. We estimated that inocula containing 1 × 10^7^, 1 × 10^6^ and 1 × 10^5^ spores/ml deliver at least one spore to 95%, 75%, and 50% of the mosquitoes sprayed, respectively. As mosquito mortality increases with inoculum load, we plotted spore counts per mosquito versus the probability of death derived from our bioassays for the WT and transgenic strains (Met-AaIT, Met-Hybrid, and Met-Hybrid/AaIT) (Fig. 4). Together, these data revealed that the lethal dose per mosquito at which 100% of mosquitoes die (LD100) is 10 spores, 8 spores, 7 spores, and 6 spores for WT, Met-AaIT, Met-Hybrid and Met-Hybrid/AaIT, respectively. The results explain the incomplete mortality seen even with our highest spore concentration (1 × 10^7^), and the complete mortality seen in mosquitoes treated through contact with oil-impregnated cloth, which delivers far more spores to every mosquito. This bodes well for oil-impregnated cloth as a delivery system for entomopathogenic fungal spores and for the translatability of spray method results to the field.

In conclusion, fungi can be genetically modified to strategically maximize their success as biocontrol agents^13, 20^. When their impact on malaria transmission is considered, transgenic fungi applied on sheets meet WHO standards for effective control of malaria within 5 days post-exposure, indicating that the inclusion of transgenic *Metarhizium* in pre-existing control efforts would effectively decrease malaria transmission. Some female mosquitoes infected with transgenic fungi will lay eggs 3–4 days after a bloodmeal, so infected mosquitoes may still pass their genes onto the next generation, but infection will prevent further oviposition. Our study emphasizes the need to consider the effect of fungi on blood feeding for modeling of existing mosquito control techniques in conjunction with transgenic *Metarhizium* ^21^.

## Methods

### Ethics statement

Ethical permissions were obtained through the Institutional Review of Institut de Recherche en Science de la Santé (IRSS) and Centre Muraz ethics committee. In addition, prior authorization was granted from the Burkina Faso National Biosecurity Agency for importing and using *Metarhizium* transgenic fungi expressing Hybrid toxin for semi-field and lab work (Supplementary File). All experiments with guinea pigs were carried out in strict accordance with the recommendations in the Guide for the Care and Use of Laboratory Animals of the National Institutes of Health. In addition, the protocols followed the IRSS Animal Welfare Assurance A5926-01. Trained personnel and veterinarians cared for all animals involved in this study.

### Mosquito colony

The study was carried out in a joint IRSS and Centre Muraz laboratory in Bobo Dioulasso, Burkina Faso. For bioassays, we used adult mosquitoes *An. coluzzii* and *An. gambiae* reared from larval collections at Vallée du Kou (11°23′N, 4°24′W) and Soumousso (11°04′N, 4°03′W), respectively. Mosquitoes from these areas are resistant to multiple insecticides^6^. The laboratory-reared Kisumu strain of *Anopheles gambiae s.s*. was used as an insecticide-susceptible reference strain. All larvae were simultaneously reared under standard, controlled conditions (26 ± 2 °C, 80 ± 10% relative humidity (RH) and 12:12 light-dark cycle). Larvae were fed daily with Tetramin™. Pupae were collected and transferred into holding cages measuring 30 cm × 30 cm × 30 cm covered with mosquito netting. Upon emergence, mosquitoes were immediately morphologically identified and sexed using a key and were maintained in cages on 6% sugar solution for 2–3 days prior to fungal infection. Only two to three day old females were used for bioassays with fungi.

### Honeybee rearing

Honeybees, a locally reared *Apis mellifera adansonii* Latreille 1804, were kept in groups of 25 to 30 in cages in an insectarium at 25.3 ± 1 °C, and fed on 10% sterile glucose with syrup of honey and sterile tap water.

### Fungal strains and fungal transformation

The African isolate of *Metarhizium pingshaense* used in this study was maintained on potato dextrose agar at 26 °C and 70% RH. This *M. pingshaense* strain was chosen because it has a narrow host range and does not infect local Burkina Faso insects *Apis mellifera adansonii* (honeybees) and *Schistocera gregaria* (locusts). Toxin genes were chemically synthesized with the MCL1 signal peptide to ensure secretion, and they were cloned in a common transfer plasmid pPK2-Bar-GFP^22^, downstream of the Mcl1 promoter as described^13^, to target high-level production of each toxin into the hemolymph. To produce the dual toxin expressing strain Met-Hybrid/AaIT, we used the plasmid pAaIT1 expressing AaIT1 described previously^13^. To construct the Ti plasmid expressing Hybrid, the cassette that contains the hybrid encoding sequence downstream of the Mcl1 was excised from the plasmid pMcl1-Hybird^13^ with EcoRI and NotI, treated with T4 DNA polymerase, and inserted into the EcoRV site of pPK2-Sur-GFP^22^ to form the Ti plasmid. The pBar-Hybrid and pSur-Hybrid plasmids were consecutively transformed into *M. pingshaense* using *Agrobacterium* ^13^ as described^15^ with Bar and Sur genes, respectively, as selection markers. The Ti plasmid also contains the gene for green fluorescent protein (GFP) under a constitutive promoter (GdpA). An RFP-expressing strain was produced as described^23^ and confirmed to show WT virulence and growth against *Anopheles*. The presence of each toxin gene in the *Metarhizium* genome was confirmed by PCR. PCR products from each of the transformants were electrophoresed on agarose gel, excised and sequenced to confirm the presence of transgenes in transformed *Metarhizium* strains. The primers used in this study and their respective usages are summarized in Supplementary Table 3.

### Fungal formulations and infection of insects in World Health Organization tubes

Fungal strains were harvested after 2 weeks of incubation at 26 °C. Five ml of sesame oil formulation at 8.0 × 10^7^ conidia ml^−1^ were used to impregnate pieces of 12 × 15 cm black cotton sheets. This produced a final density of ~2 × 10^6^ conidia/cm^2^. Control treatments were oil preparations without fungal spores. Impregnated sheets were dried for 16 h overnight at ambient temperature (26 ± 1 °C, 80 ± 10% RH) before placing inside WHO bioassay tubes (15 cm long, 4 cm diameter; Supplementary Figure 2). 25–30 mosquitoes or bees were exposed in the WHO tube for 1 h. After exposure, each group was transferred into 20 cl plastic cups for twice-daily observations of mortality and, in the case of the mosquitoes, blood feeding choice experiments (Supplementary Figure 3).

Mortality was counted twice daily. For any deaths, cadavers were surface sterilized and placed on 1.5% agar plates to promote fungal emergence and confirm if cause of death was fungal. Mortality caused by other factors was not included in survival analysis.

### Fungal growth observations

Mosquito mortality was measured twice daily. Cadavers were immediately removed from their cups and each was washed once for 20 s with 1% sodium hypochlorite for 20 s and twice with sterile distilled water for 40 s. Washed cadavers were then placed on a 1.5% agar plate. Fungal growth on mosquitoes was confirmed microscopically after 3–5 days incubation.

### PCR assay of insecticide resistance

During mortality bioassays, two randomly chosen legs from each mosquito were dissected. This subsample was then analyzed by PCR to definitively identify the species, molecular form^24^ and the *kdr* mutation levels^25^.

### Dual transgene fungal infections

Comparative transgene experiments were conducted at the University of Maryland as described^15^. Briefly, in three replicates, 30 female mosquitoes (*An. gambiae* Keele) were cold anesthetized (4 °C) and sprayed 8 times with an atomizer containing a conidial suspension in 0.01% Tween-80. Treated mosquitoes were checked daily for mortality for 11 days.

### Blood feeding choice tunnel

A 60-cm long, glass choice tunnel (25 cm × 25 cm area) was used for blood feeding assays (Supplementary Figure 1a,b). A 25-cm square of polyester netting was fitted at one end of the tunnel as a compartment. A netted barrier was placed one-third along the length of the glass tunnel separating the tubes into short and long sections. The barrier was 400 cm^2^ (20 × 20 cm), with nine 1 cm diameter holes for passage; one hole was located at the center of the square and the other eight were equidistantly located 5 cm from the border. This choice chamber is designed as a miniaturized proxy for a traditional West African home. The largest section of such a home is the veranda that serves as a sitting area. This corresponds to the first compartment of the tunnel (40 cm long). The second smaller part of a traditional house is the bedroom where residents sleep under bed netting. This corresponds to the smaller compartment of the tunnel (20 cm long). We placed the guinea pig within 5 cm of this compartment to represent a sleeping occupant at night (Supplementary Figure 1a,b). Fifty non-blood-fed female mosquitoes (*An. coluzzii*) were released into the long section of the tunnel. In this design, female mosquitoes are normally attracted through the barrier into the smaller compartment by the guinea pig to blood feed, but they cannot bite the guinea pig. In full darkness between 6 pm and 6 am, mosquitoes interested in blood feeding were free to fly through the tunnel, locate the holes and pass through them to reach the guinea pig. The location of mosquitoes after this period was recorded, and those in the section closest to the guinea pig were considered to have interest in blood feeding. Mosquitoes were removed from each section of the tunnel and counted separately.

The choice chambers were maintained at 27.34 °C average temperature (range 27.06 °C to 27.60 °C). The average relative humidity was 76.60% (range 75.90% to 77.00%) The mortality during the assay was recorded, but only live mosquitoes were considered for analysis.

### WHO Tube Cloth Spore Dose Estimation

Two protocols were used to determine the number of spores picked up per insect from cloth impregnated with ~2 × 10^6^ conidia/cm^2^ Met-Hybrid. Black cotton cloth cheaply available in Burkina Faso was used for bioassays. Paper supports were stapled outside of the cloth to maintain rigidity inside the WHO cylinders during the assay. First, 25 female mosquitoes or bees were introduced in the WHO cylinder with impregnated cloths. After 1 h of contact, insects were transferred to plastic cups for 2 h then individually washed in 200 μl of 0.05% Tween-80 by vigorously vortexing them for 5 min. The number of spores picked up by each insect was counted using an improved Neubauer haemocytometer. No fluorescent spores remained on the insects after washing.

In the second protocol used only for mosquitoes, instead of washing the mosquitoes and counting the number of spores, individual mosquitoes were crushed in 200 μl of 0.05% Tween-80, vortexed and the homogenate spread onto *Metarhizium* selective media^26^. Five days later, the number of spores per mosquito was determined by counting the colony forming units (CFUs). There were no significant differences in the number of spores per mosquito determined using these two protocols.

The second protocol was also used to count spores delivered by spraying aliquots of 20 female mosquitoes per concentration.

### Data analysis

Statistical analyses were carried out with R (version 3.2.4; Supplementary Code). Mosquitoes were considered alive if they could stand upright and dead if they were unresponsive to stimuli following the 2013 recommendations by the WHO Pesticides Evaluation Scheme^18^.

## Electronic supplementary material

Supplementary Information

Combined Datasets
