# Supplementary material for: Improved efficacy of an arthropod toxin expressing fungus against insecticide-resistant malaria-vector mosquitoes
Source: Sci Rep. 2017 Jun 13;7:3433. doi: 10.1038/s41598-017-03399-0 (PMC5469824; doi:10.1038/s41598-017-03399-0)
Supplement: Supplementary file 1 — Supplementary Information [file 41598_2017_3399_MOESM1_ESM.pdf]

Title: Improved efficacy of an arthropod toxin expressing fungus against insecticide-resistant malaria-vector mosquitoes

Etienne Bilgo[1], Brian Lovett[2], Weiguo Fang[3] Niraj Bende[4], Glenn F. King[4], Abdoulaye Diabate[1] and Raymond J. St. Leger[2*]

[1]Institut de Recherche en Sciences de la Santé/Centre Muraz, Bobo-Dioulasso, Burkina Faso; [2]Department of Entomology, University of Maryland, College Park, Maryland 20742, USA; [3]Institute of Microbiology, College of Life Sciences, Zhejiang University, Hangzhou, Zhejiang 310058, China; [4] Institute for Molecular Bioscience, The University of Queensland, St. Lucia, QLD 4072, Australia

* To whom correspondence should be addressed:

Raymond J. St. Leger

E-mail: stleger@umd.edu

Tel: +1(301)405-5402

Fax: +1(301)314-9290

Supplementary Information

Supplementary Figure 1

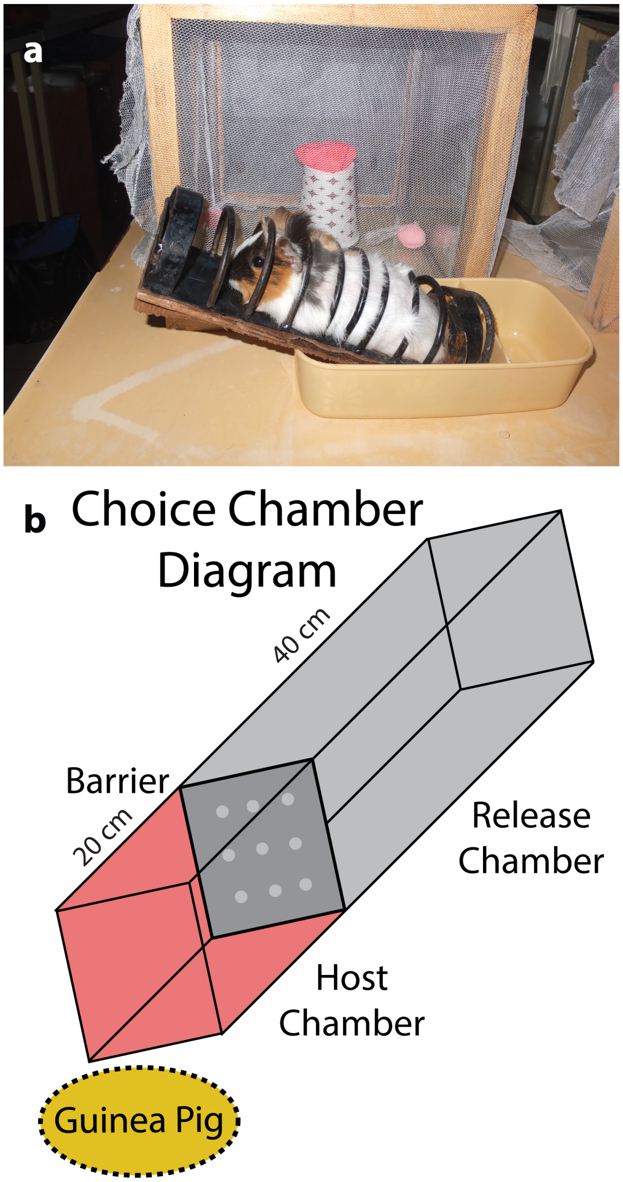

Supplementary Figure 1 Legend: (a) Configuration for experiments testing host-seeking behavior using guinea pigs and a tunnel choice chamber with nine small holes cut into a barrier between compartments. **(b)** A diagram representing dimensions of the guinea pig choice chamber approximating a traditional layout for a Burkinabe house.

Supplementary Table 1

	RR	RS	SS	Fq (R)	Number of mosquitoes
An. coluzzii	98.3%	0	1.7%	98.3%	173
***An. gambiae* s.s.**	92.9%	0	7.1%	92.9%	70

Supplementary Table 1 Legend: Insecticide resistance in wild-caught mosquitoes, *An. coluzzii* and *An. gambiae* s.s. resistance levels to pyrethroid insecticides as measured by PCR testing for Knockdown (kdr) resistance. RR, RS, and SS represent alleles found for insecticide sensitivity (denoted by S) and insecticide resistance (denoted by R); Fq is the frequency of resistance.

Supplementary Table 2

Treatment	Day	LC50 (Mean ± SE)	Expected	Ratio (Obs/Exp)
Hybrid/AaIT	4.5	6.72E+06 ± 8.39E+05	1.33E+07	0.504
Hybrid/AaIT	5	4.62E+06 ± 1.06E+06	1.03E+07	0.447

Supplementary Table 2 Legend: 5 day LC50 and expected 5 day LC50 of stacked toxin *Metarhizium* strain calculated in accordance with methods reported by Tabashnik (Tabashnik,1992). The ratio reported is the observed LC50 of the dual toxin strain over the expected LC50: a value of 1 indicates no interaction, <1 indicates synergism, and >1 indicates antagonism.

Supplementary Table 3

Primers	Sequences	Usage of Primers
pMcl1-5	TAA TTC GTT CCT GGC TCA AAT TCT TTT C	For amplifying arthropod toxins successfully transformed into *Metarhizium*
TtrpC-3	TCA AGC TGT TTG ATG ATT TCA GTA ACG	

Supplementary Table 3 Legend: PCR primers for amplification of toxin sequences flanked by the *Metarhizium* Mcl1 promoter and *Aspergillus* TrpC terminator that were transformed into *Metarhizium pingshaense* genome.

Supplementary Figure 2

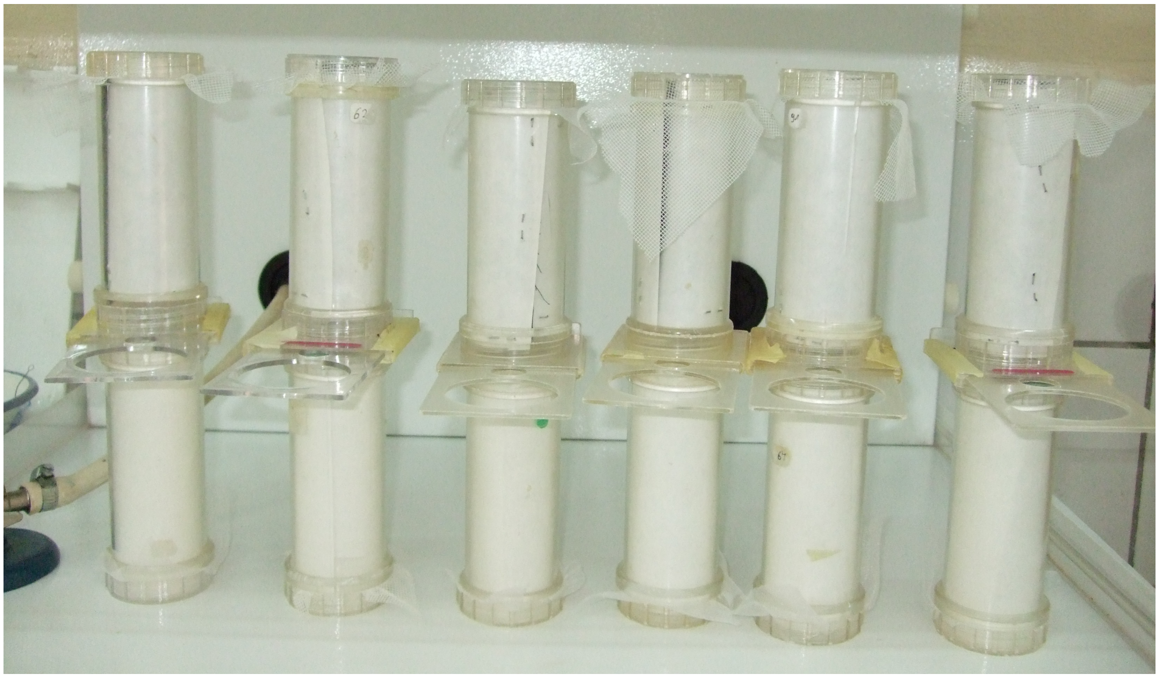

Supplementary Figure 2 Legend: WHO Mosquito Bioassay Tubes used to infect mosquitoes. Black cloth impregnated with spores was stapled to a white paper support and rolled into each bioassay tube for treatment. After treatment, mosquitoes were transferred into the empty lower tube and then placed in cups for mortality observations.

Supplementary Figure 3

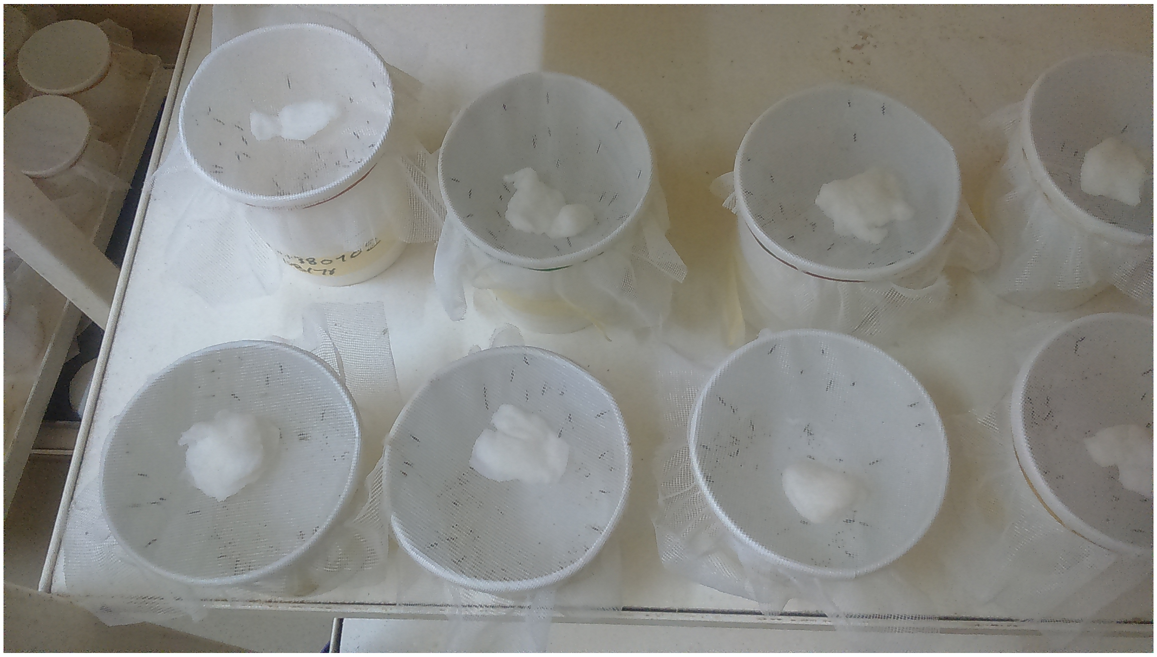

Supplementary Figure 3 Legend: Culturing cups for mortality observations. Each cup is fitted with mosquito netting and an elastic band. Cotton balls were saturated with 6% sucrose *ad libitum* for the duration of the observations.

Supplementary Code

The supplemental R script for all analyses in this manuscript is available at https://github.com/lovettbr/improved-efficacy.git
